# Supplementary figures and images for: A human mitofusin 2 mutation can cause mitophagic cardiomyopathy
Source: eLife. 2023 Nov 1;12:e84235. doi: 10.7554/eLife.84235 (PMC10619978; doi:10.7554/eLife.84235)

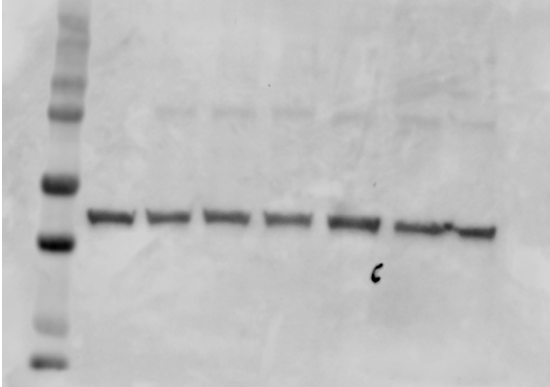

Supplement: Figure 1—source data 2. [file elife-84235-fig1-data2.zip › Figure 1 source data 2/Figure 1 source data 2 bactin.png]

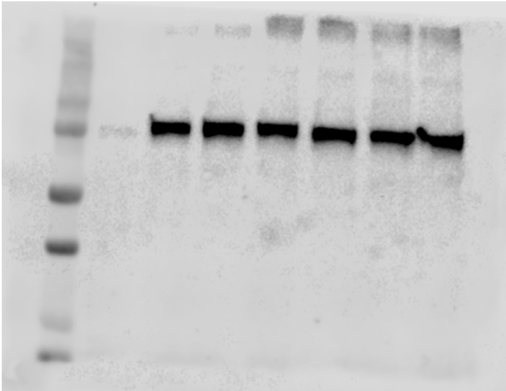

Supplement: Figure 1—source data 2. [file elife-84235-fig1-data2.zip › Figure 1 source data 2/Figure 1 source data 2 mfn2.png]

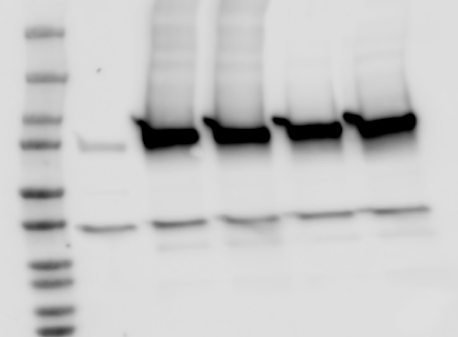

Supplement: Figure 6—source data 1. [file elife-84235-fig6-data1.zip › Figure 6 source data1 /Figure 6 source data 1.png]
